# YOLO-ALW: An Enhanced High-Precision Model for Chili Maturity Detection

**DOI:** 10.3390/s25051405

**Published:** 2025-02-25

**Authors:** Yi Wang, Cheng Ouyang, Hao Peng, Jingtao Deng, Lin Yang, Hailin Chen, Yahui Luo, Ping Jiang

**Affiliations:** 1 College of Information and Intelligence, Hunan Agricultural University, Changsha 410128, China; wangyi@hunau.edu.cn (Y.W.); ouyang@stu.hunau.edu.cn (C.O.); sx20230201@stu.hunau.edu.cn (H.P.); 984711035@stu.hunau.edu.cn (J.D.); 2767959117@stu.hunau.edu.cn (L.Y.); 1152400900@stu.hunau.edu.cn (H.C.); 2 College of Mechanical and Electrical Engineering, Hunan Agricultural University, Changsha 410128, China; luoyh@hunau.edu.cn

**Keywords:** deep learning, fruit detection, attention mechanism, pepper maturity recognition, YOLOv8n

## Abstract

Chili pepper, a widely cultivated and consumed crop, faces challenges in accurately determining maturity due to issues such as occlusion, small target size, and similarity between fruit color and background. This study presents an enhanced YOLOv8n-based object detection model, YOLO-ALW, designed to address these challenges. The model introduces the AKConv (Alterable Kernel Convolution) module in the head section, which adaptively adjusts the convolution kernel shape and size based on the target and scene, improving detection performance under occlusion and dense environments. In the backbone, the SPPF_LSKA (Spatial Pyramid Pooling Fast-Large Separable Kernel Attention) module enhances the integration of multi-scale features, facilitating accurate differentiation of peppers at various maturity stages while maintaining low computational complexity. Additionally, the Wise-IoU (Wise Intersection over Union) loss function optimizes bounding box learning, further improving the detection of peppers in occluded or background-similar scenarios. Experimental results demonstrate that YOLO-ALW achieves a mean average precision (mAP_0.5_) of 99.1%, with precision and recall rates of 98.3% and 97.8%, respectively, outperforming the original YOLOv8n by 3.4%, 5.1%, and 9.0%, respectively. Grad-CAM feature visualization highlights the model’s improved focus on key fruit features. YOLO-ALW shows significant promise for high-precision chili pepper detection and maturity recognition, offering valuable support for automated harvesting applications.

## 1. Introduction

Chili pepper is a crucial agricultural product globally, with its cultivation and consumption being widespread. Precise determination of chili maturity is vital for both agriculture and the food processing industry. In agriculture, it guides optimal harvesting, ensuring peppers have the best flavor, nutrition, and market value. For instance, overripe peppers are more likely to spoil, while underripe ones may lack flavor. In food processing, it helps maintain product quality consistency. However, accurately detecting chili maturity is difficult due to occlusion, small target size, and color similarity with the background.

Convolutional Neural Networks (CNNs), as a crucial branch of deep learning, have recently seen preliminary explorations in the area of fruit maturity detection. For example, Lin et al. [1] developed a leaf color-parameter maturity determination model using multivariate regression and BP neural networks to effectively identify tobacco leaf maturity under different environmental conditions. Agung et al. [2] employed chili pepper image data and the backpropagation method, combined with a MATLAB application, to successfully identify the maturity levels of chili peppers, achieving an accuracy rate of 97.78% in training and testing datasets. Hendrawan et al. [3] divided the maturity stages of large green peppers into three levels (Maturity Stage 1: Maturity Index 1/34 days, Stage 2: Maturity Index 3/47 days, Stage 3: Maturity Index 5/60 days), and achieved a 93.89% accuracy using GoogLeNet with an SGD optimizer. Fan et al. [4] improved the YOLOv5s model for lotus pod maturity detection and deployed the model on a Raspberry Pi 4B, enabling efficient maturity detection. Zainudin et al. [5] classified chili peppers into immature, moderately mature, and mature stages, utilizing CNN for modeling and achieving an 85% recognition accuracy. Trieu et al. [6] proposed an automatic classification system for dragon fruit, which achieved an accuracy of over 96%, effectively addressing the evaluation and classification issues of dragon fruit. Li et al. [7] introduced a lightweight YOLOv5s model for detecting dragon fruit under varying light conditions, demonstrating the effectiveness of YOLO models in fruit detection tasks.

In terms of imaging technology, Li et al. [8] introduced a dual-frequency lidar compressed sensing 3D imaging technology based on full-phase fast Fourier transform, which provides more accurate spatial information and addresses challenges like occlusion and small object detection in chili pepper recognition. Chen et al. [9] proposed dynamic visual servo control methods for fruit-picking robots, enabling continuous operation in orchards and offering potential solutions for automated chili pepper harvesting.

Notably, attention mechanisms have shown significant potential in fruit maturity detection [10,11,12,13,14,15,16,17]. In oil tea maturity detection, Chen et al. [18] improved the YOLOv7 model by incorporating an attention mechanism, enhancing recognition accuracy by focusing on the unique appearance and growth environment characteristics of the fruit. For blueberry ripeness recognition, Wang Lishu and Li Zhu et al. [19,20] improved the recognition accuracy by using the improved YOLOv4-Tiny model and VGG16 neural network combined with the attention mechanism, respectively, with full consideration of the size, color distribution, and other features of blueberry fruits. In apple maturity detection, Zhang et al. and Yuan et al. [21,22] combined improved YOLOv3 and joint transfer learning with adaptive learning rates, integrating attention mechanisms to achieve more precise recognition under natural scene conditions with varying maturity states and complex backgrounds. In citrus maturity research, Wang, C. et al. [23] combined image processing with deep learning, integrating the R-LBP algorithm and YOLO-CIT model with an attention mechanism, focusing on texture and color features to improve detection performance. In strawberry maturity detection, Tao, Z. and Youchen, F. et al. [24,25] achieved efficient recognition based on the improved YOLOv5 model, combined with techniques such as dark channel enhancement and attention mechanisms for strawberry color and shape variations and the complexity of the growing environment, respectively. For coconut maturity recognition, Kannan, R.M. et al. [26] utilized an ensemble of fuzzy logic and deep learning models with an attention mechanism to highlight maturity features and improve detection accuracy.

In chili pepper detection, Li et al. [27] integrated YOLOv4-Tiny with an adaptive spatial feature pyramid method, incorporating the CBAM attention mechanism and multi-scale prediction, which significantly improved performance in detecting occluded and small targets, achieving 96.91% accuracy with a detection time of 11.24 ms per image. However, the model faces limitations under varying lighting conditions and complex backgrounds, and requires substantial computational resources during deployment. Despite considerable progress in fruit maturity detection, challenges remain in chili pepper maturity detection. Pepper plants often have dense foliage, causing severe occlusion, and immature fruits have similar colors to the background under different lighting conditions, leading to misclassification.

To address the challenges in chili pepper maturity detection, this paper proposes an improved YOLOv8n-based method. The approach introduces the AKConv module for adaptive convolution kernel adjustment, enhances feature extraction with the SPPF_LSKA module, and replaces the C-IoU loss with the Wise-IoU loss to improve detection accuracy, especially in occluded or complex environments. This paper is organized as follows: Section 2 details the dataset, maturity classification standards, and the YOLO-ALW model construction. Section 3 presents experimental evaluations, including ablation studies and comparisons. Section 4 discusses model improvements, performance stability, and dataset optimization. Section 5 concludes with a summary of the results and the model’s potential for practical applications.

## 2. Materials and Methods

### 2.1. Dataset

A total of 1456 images of chili peppers at different maturity stages were collected for this study. The data collection process was carried out in four separate sessions, each conducted at different chili pepper cultivation bases in Changsha City, Hunan Province, China. In each session, more than 300 chili peppers were photographed. The image dataset includes chili pepper data captured at different times of the day—morning, noon, and afternoon—with specific details regarding the collection times and locations provided in Table 1. The weather conditions during data collection included both sunny and cloudy days. The images were taken from various angles, including top–down, side, and upward perspectives, with different lighting conditions employed, such as backlighting, front lighting, and side lighting.

To address the potential complexities encountered during chili pepper harvesting, the dataset includes images with varying levels of lighting conditions, fruit overlap, and leaf occlusion. Additionally, the dataset contains chili peppers at different maturity stages, with single images featuring peppers of the same type or mixed classes to meet the requirements of chili pepper harvesting. The specific shooting conditions are illustrated in Table 2. Among the collected peppers, 2500 are at the mature stage, 2756 are overripe, and 935 are immature. “Level shot” refers to images captured at a horizontal angle relative to the peppers, while “top–down shot” is taken with a downward tilt of 60–80 degrees, and “upward shot” is taken with an upward tilt of 60–80 degrees. The overlap scenarios refer to cases where some of the peppers are occluded in the images, categorized into two levels of occlusion: 25% and 50%.

**Figure 1:**
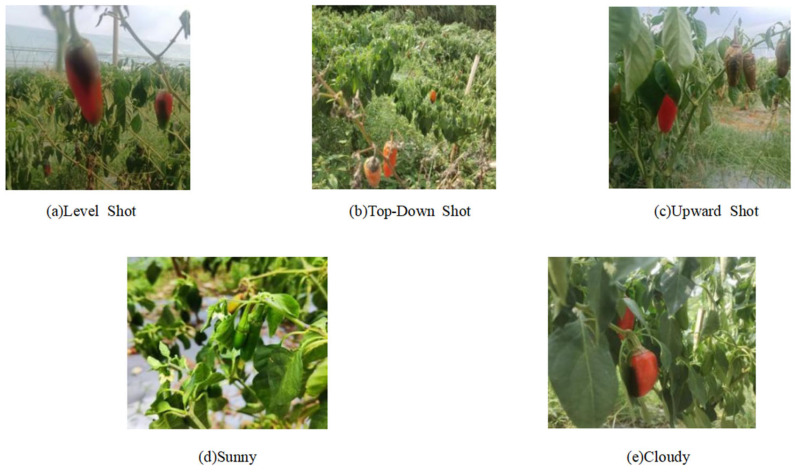
Chili pepper images under different shooting angles and weather conditions.

The images in the dataset were all captured using mobile phones sourced from Changsha, Hunan Province, China. The images were stored in two aspect ratios, 4:3 and 1:1. Due to the use of different phone models for image capture, the dataset contains images with varying resolutions, including 4032 × 3024, 3024 × 3024, 3072 × 3072, and 3000 × 3000 pixels, thereby providing inputs at different pixel scales. A sample image from the dataset is shown in Figure 1.

The quality and diversity of data samples play a crucial role in the performance of models for chili pepper harvesting. Due to variations in the maturation times of different chili pepper varieties and the uncertainties of harvesting schedules, data collection through field photography is often constrained by weather conditions and requires substantial human and material resources. Therefore, the use of a large-scale sample dataset is essential to enhance the model’s generalization ability and robustness. A diverse dataset not only aids the model in learning relevant features and improving accuracy but also provides additional unknown information, which helps strengthen the model’s adaptability in real-world environments [28,29,30]. Furthermore, appropriate image preprocessing techniques, such as brightness adjustment, mirroring, translation, Gaussian noise addition, cropping, and rotation, can effectively reduce the risk of overfitting and enhance the model’s generalization performance, thus meeting the demands of various tasks and models. To improve the practical applicability of the chili pepper harvesting model, this study augmented the chili pepper image dataset to 2385 images and partitioned it into training and test sets in a 2000:385 ratio. The data augmentation techniques not only increased the sample size but also ensured a balanced distribution of chili peppers at different maturity stages by manually selecting the data. This approach mitigates potential model bias due to data imbalance and ensures the representativeness of the training data and the effectiveness of the model.

### 2.2. Chili Pepper Maturity Classification

In the field of agriculture, the classification of chili pepper maturity is a highly significant research topic, with profound implications for both the economic benefits of producers and the practical operations involved. Accurately defining the maturity stages of chili peppers provides a scientific foundation for the entire agricultural process, including production, harvesting, and market operations. Given that chili peppers are a key global agricultural commodity widely cultivated and consumed, the differences in flavor, taste, and nutritional content at various maturity stages are substantial. Therefore, precise determination of chili pepper maturity is crucial in both the agricultural and food processing industries. Currently, there is a lack of publicly available datasets for chili pepper maturity, and the dataset constructed in this study aims to fill this gap [31].

Traditional methods for determining chili pepper maturity have involved various indicators. Some studies have explored the use of vitamin C content, peel hardness, and pigment levels as potential criteria for assessing chili maturity, although this is not the sole or universal approach. With the advancement of agricultural technologies, cutting-edge techniques such as image processing, spectral analysis, and infrared imaging have been widely applied in the field of chili pepper maturity determination, significantly enhancing the objectivity and accuracy of assessments and improving production efficiency [32,33,34]. In this study, based on expert experience, maturation time, and fruit appearance characteristics, chili peppers were classified into three categories: mature, immature, and overripe. The detailed classification criteria are presented in Table 3. The labeled images were then used for large-scale training through deep learning techniques, with the aim of achieving efficient, convenient, and rapid chili pepper maturity classification.

**Figure 2:**
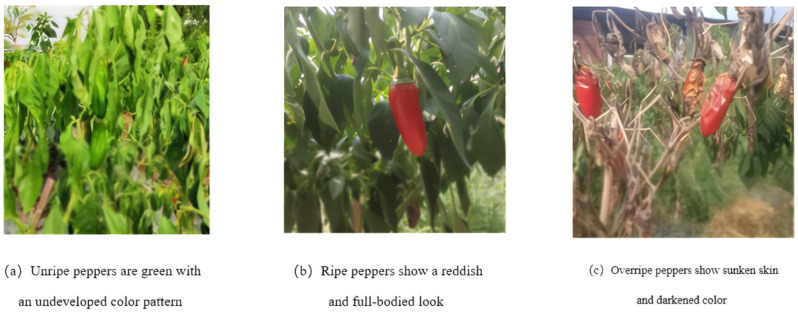
Categorization of maturity.

In this paper, based on the actual condition of pepper picking, the maturity of peppers was classified into 3 categories, which were overripe fruits, surface-ripe fruits, and unripe peppers. The maturity classification of chili peppers is shown in Figure 2. Figure 2a presents the unripe pepper fruits, which are overall green in color, and the color pattern is not yet fully expanded. Figure 2b presents an example of a fully ripe chili pepper fruit with a reddish surface and a full fruit. Figure 2c shows an overripe fruit with sunken skin and darkened color. Fruits were classified as ripe if the surface was 100% fully ripe and contained a high degree of red pigmentation; classified as unripe if they were less than 100% fully ripe and had a greenish color; and classified as overripe if there was a sunken surface along with a high degree of red pigmentation. Peppers with different levels of maturity were manually labeled in the finished pepper dataset.

**Figure 3:**
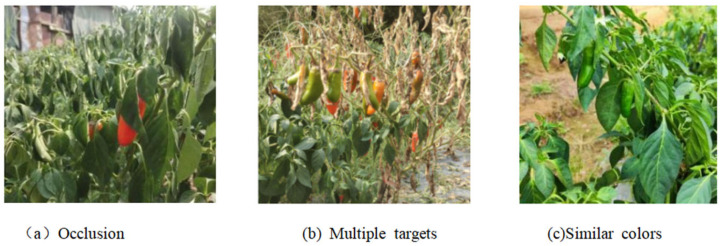
Data presentation in different environmental conditions.

The different scenarios of pepper photography are shown in Figure 3. These include overlapping fruits, fruits with occlusion by branches and leaves, fruits with light and shadow on the surface, fruits without light and shadow on the surface, and fruits with and without debris. Also, the images produced for the chili pepper dataset include single-target and multi-target scenarios, fruits with or without overlapping, and fruits with or without occlusion by branches and leaves.

### 2.3. Pepper Maturity Detection Model and Improvement

#### 2.3.1. YOLO-ALW Network Construction

The YOLOv8 architecture, known for its real-time object detection performance, uses a CSPDarknet53 backbone with the C2f module, which replaces the traditional CSPLayer to improve feature extraction and small object detection. Its Neck incorporates the Spatial Pyramid Pooling Fast (SPPF) layer, which speeds up computation through fixed-size feature maps, enabling efficient multi-scale detection. The decoupled Head design separately handles objectness, classification, and regression tasks, enhancing detection accuracy.

**Figure 4:**
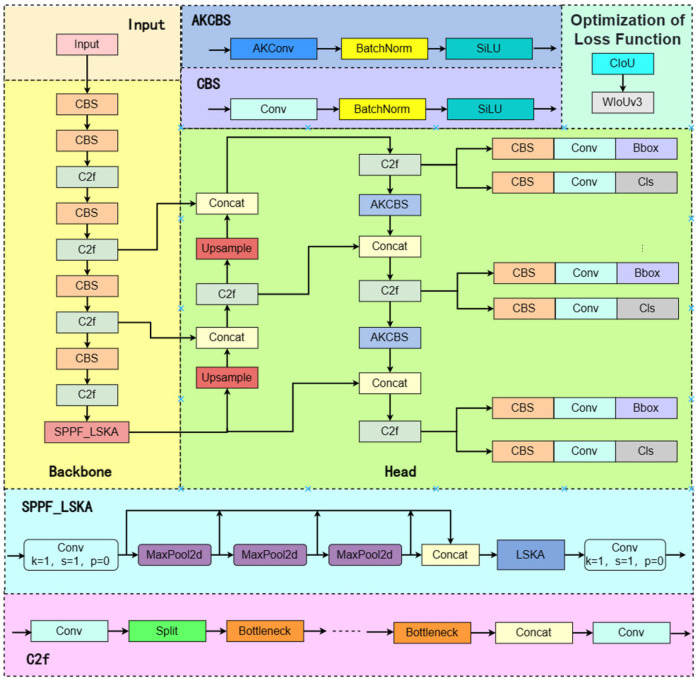
The network structure of YOLO-ALW.

In the context of chili pepper detection, challenges such as vigorous plant growth, dense foliage occlusion, and the similarity in color between immature fruits and the background [35,36] prompted a series of optimizations to the YOLOv8n model in this study. First, the Head section introduced the AKConv module, which adaptively adjusts the shape and size of convolution kernels to enhance the model’s ability to handle diverse and complex targets. Second, the Backbone was enhanced with the SPPF_LSKA module, optimizing the integration of multi-scale features and improving the model’s capacity to capture contextual information in chili pepper images, thereby more effectively distinguishing chili peppers at various maturity stages. Finally, to address the limitations of the traditional C-IoU loss function, this study employed the Wise-IoU (WIoU) loss function, which incorporates a dynamic non-monotonic focusing mechanism. This function adjusts the gradient gain distribution based on the quality of anchor boxes, optimizing the learning process for samples of varying quality and ultimately improving the model’s localization accuracy and overall performance. These improvements significantly enhanced the model’s performance in chili pepper maturity detection, particularly in handling complex occlusion and background interference, demonstrating greater robustness and higher precision. The structure of the improved network model is shown in Figure 4.

#### 2.3.2. Alterable Kernel Convolution (AKConv)

Conventional convolutional modules use fixed kernel shapes and sizes for feature extraction, which may not effectively capture the diversity of variations in pepper shapes, sizes, and ripeness. The AKConv module [37] addresses this by allowing flexible kernel shapes and sizes, with adjustable parameters that enable the network to adapt to target variations. This flexibility enhances network performance by improving feature extraction, particularly for irregularly shaped objects like peppers.

**Figure 5:**
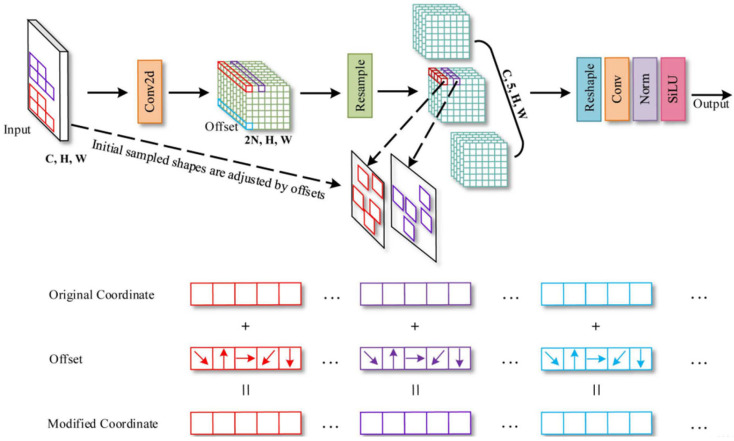
Schematic diagram of the AKConv module.

In the actual convolution operation process, first, for any given size of the convolution kernel, AKConv generates the initial sampling coordinates P_n_ through a specific algorithm, where the parameter n indicates the size of the convolution kernel, and then the algorithm, based on the value of n as well as the preset rules, determines the distribution of the sampling coordinates, and after that, for each position P_0_ in the image, AKConv is convolved with the parameter w with (P_0_ + P_n_) convolution operation, thus realizing the extraction of local features. In order to make the convolution kernel better adapt to the change in target shape, AKConv also introduces the offset Offset, which can capture the shape information and position change in the target in the image by learning in the network; that is to say, before performing the convolution operation, the offset Offset obtained by learning is added with the original sampling coordinates P_n_ to obtain the modified sampling coordinates (P_0_ + P_n_ + Offset). In this way, the positions of the sampling points can be dynamically adjusted according to the shape of the target, so that the convolution kernel can more accurately cover the region related to the target and extract more representative and targeted features. The AKConv schematic is shown in Figure 5.

#### 2.3.3. Integration of Separable Large Kernel Convolution Attention Mechanism

The Spatial Pyramid Pooling Fast (SPPF) module, as an effective spatial feature aggregation method, captures feature information at different levels through pooling operations at multiple scales, providing rich multi-scale representations for subsequent detection tasks. However, when processing chili pepper images in complex scenes, issues such as insufficient focus on local information and limitations in receptive field size may arise. To address these challenges, we introduce the Large Separable Kernel Attention (LSKA) module.

The LSKA module decomposes the traditional 2D depthwise separable convolution kernel [38] into two cascaded 1D convolution kernels, effectively reducing the computational and memory requirements of the model. This decomposition enables the network to leverage large convolution kernels to capture long-range dependencies within the image, while avoiding the quadratic growth in parameters and computational complexity that would occur with increasing kernel sizes. Specifically, the LSKA module first decomposes a K × K convolution into a (2d − 1) × (2d − 1) depthwise convolution (DW-Conv), a K/d × K/d depthwise dilated convolution (DW-D-Conv), and a 1 × 1 convolution (Conv). Furthermore, the depthwise convolution and the depthwise dilated convolution are further separated into horizontal and vertical components. Finally, the convolution features are combined using the Hadamard product operation, allowing for fine-tuning of the input features and enhancing the model’s performance and efficiency across various visual tasks.

**Figure 6:**
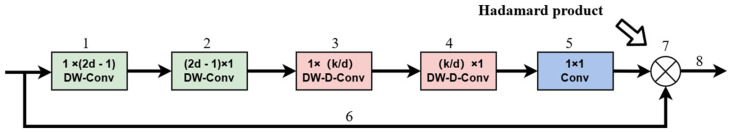
Structural diagram of the LSKA module.

This design allows the model to more effectively handle large-scale chili pepper image data and provides scalability for handling extremely large kernels, thereby accommodating chili targets of different sizes and shapes. The structure of the LSKA module is shown in Figure 6.

The fused SPPF_LSKA module introduces the Large Separable Kernel Attention (LSKA) mechanism on top of the traditional Spatial Pyramid Pooling (SPPF), which significantly enhances the feature extraction capability. The module first captures multi-scale and multi-level spatial features through successive convolutional operations and a maximum pooling layer, followed by concatenating these features in channel dimensions through a Concat layer, which enhances the feature diversity. Then, the LSKA module performs adaptive feature recalibration with a 1 × 1 convolutional kernel, where the parameter k represents the size of the convolutional kernel, s represents the step size, and *p* represents the padding, all of which are set to 1 here, which ensures that the spatial dimensionality of the feature map remains unchanged, while enhancing the expressiveness of the features through the attention mechanism.

**Figure 7:**
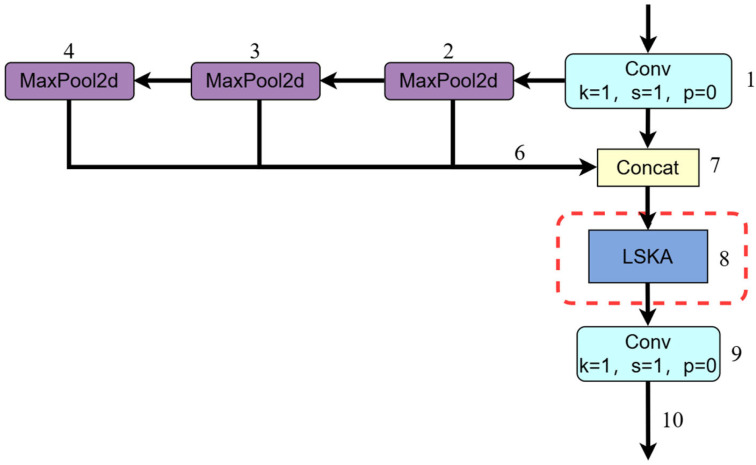
Structural diagram of the SPPF_LSKA module.

The fused module not only retains the detection advantage of the SPPF module for targets of different sizes, but also further enhances the model’s ability to capture key features through the attention weighting of the LSKA module, especially when the target is partially occluded or the background is interfered with. The structure of the SPPF_LSKA module is depicted in Figure 7.

#### 2.3.4. Bounding Box Regression Loss Function Optimization for Dynamic Focusing Mechanisms

In natural environments, chili fruits at the unripe stage often exhibit colors that closely resemble the background, posing significant challenges to model recognition. Additionally, chili fruits are typically arranged in clusters on the same inflorescence, resulting in smaller fruit sizes and increased potential for mutual occlusion. These factors severely impact the accuracy of object detection models.

To address these issues, we introduce the Wise-IoU (WIoU) [39] loss function, which incorporates a dynamic focusing mechanism for boundary box regression. Specifically, WIoUv1 constructs an attention-based boundary box loss, while WIoUv2 and WIoUv3 further enhance WIoUv1 by introducing a gradient gain mechanism to apply an additional focusing effect. The version used in this study is WIoUv3, which improves the traditional Intersection over Union (IoU) loss by incorporating the consideration of the distance between candidate box centers. This approach allows the model to more effectively allocate gradient gains, emphasizing anchor boxes of average quality while reducing competition from high-quality anchors and harmful gradients from low-quality samples. As a result, the model’s ability to detect chili fruits with mutual occlusion is significantly improved. The computation formula for the WIoUv3 loss function is as follows.

In Equations (1)–(3), the loss value of WIoUv3 is denoted by $L_{WIoUv3}$, and the dynamic non-monotonic focusing coefficient is represented by r. Through the dynamic non-monotonic focusing mechanism, it adjusts the gradient gain according to the deviation degree β of the anchor box, thereby enabling WIoU to focus on anchor boxes of ordinary quality and enhancing the overall performance and generalization ability of the model. The smaller the value of β, the higher the overlap between the anchor box and the target box, indicating a higher quality of the anchor box. The asterisk (*) in $L_{IoU}^{*}$ represents the $L_{IoU}$ value that does not participate in backpropagation, and ${\bar{L}}_{IoU}$ represents the exponential running average value of $L_{IoU}$. Both δ and α represent hyperparameters for controlling the focusing coefficient. When β approaches δ, r approaches 1, meaning that high-quality anchor boxes will obtain a higher gradient gain; on the other hand, when β is much smaller than δ or much larger than α, r approaches 0, implying that low-quality anchor boxes will obtain a lower gradient gain, thereby reducing their impact on model training.

(1) $$L_{WIoUv3}=rL_{WIoUv1}$$

(2) $$r=\frac{\beta }{\delta \alpha^{\beta -\delta }}$$

(3) $$\beta =\frac{L_{IoU}^{*}}{{\bar{L}}_{IoU}}\in [0,+\infty )$$

In Equation (4), the width and height of the overlapping region are represented by $W_{i}$ and $H_{i}$, respectively, and the area of the union is denoted by $S_{u}$. $L_{IoU}$ is an indicator for measuring the degree of overlap between the anchor box and the target box. It is achieved by calculating the ratio of the area of the anchor box to the area of the union of the anchor box and the target box. The higher this ratio, the lower the degree of overlap between the anchor box and the target box, and vice versa. $L_{IoU}$ is the basis for calculating β and $L_{WIoUv1}$.

(4) $$L_{IoU}=1-IoU=1-\frac{W_{i}H_{i}}{S_{u}}$$

In Equation (5), *x* and *y* represent the center coordinates of the anchor box, $x_{gt}$ and $y_{gt}$ represent the center coordinates of the target box, and $W_{g}^{2}$ and $H_{g}^{2}$ represent the dimensions of the minimum bounding box. The gradient gain is adjusted by calculating the Euclidean distance between the center point of the anchor box and the center point of the target box and comparing it with the dimensions of the minimum bounding box. The larger the value of $R_{WIoU}$, the farther the distance between the center points of the anchor box and the target box, and the lower the attention of the model to such anchor boxes during the training process, thereby reducing their impact on training.

(5) $$R_{WIoU}=exp\left(\frac{{\left(x-x_{gt}\right)}^{2}+{\left(y-y_{gt}\right)}^{2}}{{\left(W_{g}^{2}+H_{g}^{2}\right)}^{*}}\right)$$

It can be seen that the WIoU loss function, through its advanced dynamic focusing mechanism and fine-tuning of the bounding box regression, is able to solve the problems of small target detection, color similarity, cluster arrangement, and occlusion in pepper ripeness detection, as well as to improve the generalization ability and robustness of the model.

## 3. Experiment and Analysis

### 3.1. Experimental Environment Configuration

The computational experiments in this study were conducted on a Lenovo laptop sourced from Changsha, Hunan Province, China. The computer equipped with an AMD Ryzen 7 7840H processor with Radeon 780 M Graphics, 16 GB of RAM, and an NVIDIA GeForce GTX 4060 GPU with 8 GB of video memory. The operating system used was Windows 11, and the deep learning framework employed was PyTorch 2.2.2, with Python 3.9 as the programming language. The computation architecture was CUDA 12.2. The model training parameters are presented in Table 4.

### 3.2. Model Training Process

In this study, the model was trained for a total of 100 epochs, with parameter updates performed based on a batch size of 16 samples per iteration. The initial learning rate was set to 0.01, which determined the step size for parameter updates during the early stages of training.

**Figure 8:**
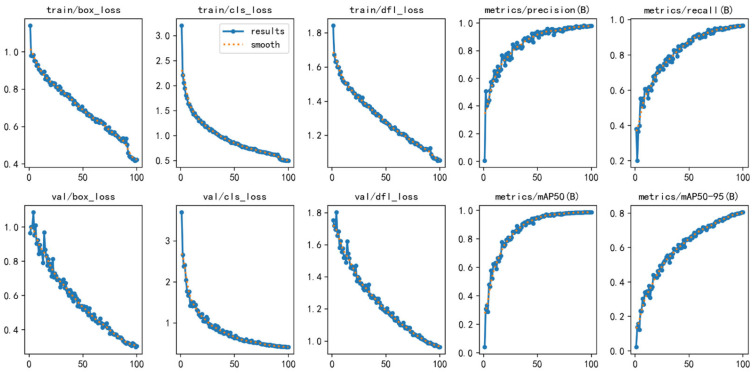
Model training and validation metrics.

The training curves (as shown in Figure 8) reveal distinct trends in the changes in box_loss, cls_loss, and dfl_loss as the number of epochs increased. Initially, all three loss values were relatively high, but they progressively decreased as the training proceeded. Specifically, box_loss decreased from approximately 3.0 at the beginning of training to nearly 1.0 by the end, indicating improved accuracy in the model’s boundary box predictions for pepper targets. Similarly, cls_loss reduced from around 1.8 to approximately 0.8, reflecting the model’s continuous improvement in classifying pepper maturity. Additionally, dfl_loss gradually decreased from a higher value to around 0.2, suggesting a reduction in the model’s discretization errors in bounding box regression.

The loss trends on the validation set also exhibited a steady decline, with val/box_loss, val/cls_loss, and val/dfl_loss decreasing progressively and showing minimal fluctuations. This indicates that the model demonstrated strong generalization ability on the validation set, with no evident signs of overfitting.

Furthermore, the model demonstrated a consistent improvement across other performance metrics. As training progressed, metrics such as precision (B), recall (B), mAP_50_ (B), and mAP_50-95_ (B) all exhibited upward trends. Initially, both precision and recall were relatively low, but with continued training, these metrics gradually increased and stabilized, ultimately reaching values exceeding 0.9 and approaching 1.0. This indicates a significant enhancement in the model’s accuracy for detecting pepper targets and its ability to identify true positive instances. Similarly, mAP_50_ (B) and mAP_50-95_ (B) also showed progressive improvement, with mAP_50_ (B) achieving a high value and mAP_50-95_ (B) exhibiting a notable increase. These results further confirm that the model’s comprehensive detection performance has been effectively optimized across different Intersection over Union (IoU) thresholds.

In summary, the model exhibited excellent convergence and stability throughout the training process, with continuous reductions in loss functions and significant improvements in precision and recall. These findings indicate that the model demonstrates effective training performance and strong learning capability for pepper maturity detection tasks.

### 3.3. Model Evaluation Metrics

In order to comprehensively evaluate and analyze the performance of the chili maturity detection model in terms of model compactness, accuracy, and real-time capability, several key metrics were selected for a multidimensional assessment. For model compactness, the evaluation primarily focused on specific indicators such as the number of parameters (Parameters), floating-point operations (FLOPs), and model size (Model Size). In terms of model accuracy, the key evaluation metrics included precision (P), recall (R), and mean average precision (mAP). The mAP was computed by averaging the precision at an Intersection over Union (IoU) threshold of 0.5, denoted as mAP_0.5_. The calculation formulas for these metrics are presented in Equations (6)–(9).

(6) $$P=\frac{T_{P}}{T_{P}+F_{P}}\times 100\%$$

(7) $$R=\frac{T_{P}}{T_{P}+F_{N}}\times 100\%$$

(8) $$AP=\int_{0}^{1}P\times dR$$

(9) $$mAP=\frac{1}{N}\sum_{i=1}^{N}{AP}_{i}$$

In the formula, $T_{P}$ represents the number of positive samples correctly identified as positive by the model, $F_{P}$ denotes the number of positive samples that the model fails to detect, and $F_{N}$ indicates the number of negative samples incorrectly classified as positive by the model [40]. N represents the total number of categories, which, in this case, is 3.

### 3.4. Experimental Results

#### 3.4.1. Ablation Experiment

To evaluate the practical effectiveness of the proposed method for chili maturity detection, an ablation study was conducted using YOLOv8n as the baseline network, with modifications made to the model. A total of eight experimental configurations were tested, each using the same experimental setup and parameters. The experimental results are presented in Table 5.

The results demonstrate that the incorporation of the AKConv module led to a significant improvement in model performance, with mAP_0.5_ increasing from 95.7% to 97.7%, precision rising from 93.2% to 96.9%, and recall improving from 88.8% to 94.3%. These substantial gains suggest that the AKConv module, through its flexible convolution kernel design, enhanced the feature extraction capability, thereby improving the accuracy and robustness of object detection. The introduction of the SPPF_LSKA module also contributed to performance improvement, although the increase was relatively modest. This may be attributed to its multi-scale feature fusion, which enhanced the model’s contextual awareness. The Wise-IoU module optimized the model’s learning process by reducing the negative impact of low-quality samples, resulting in an improvement in mAP_0.5_ from 95.7% to 97.4%, thereby demonstrating an enhancement in the model’s generalization ability. Ultimately, when all modules were integrated into YOLOv8n, the model achieved an mAP_0.5_ of 99.1%, a 3.4% increase over the original model, with precision and recall improving by 5.1% and 9.0%, respectively. These results indicate that the model’s performance reached its optimal level.

It can be seen that each module enhances the detection ability of the model to a certain extent, and there is good synergy between them, and this comprehensive optimization makes the model better able to deal with the task of target detection in complex scenarios in practical applications, thus improving the reliability and stability of the overall system.

#### 3.4.2. Contrast Experiment

To validate the advantages of the YOLO-ALW model proposed in this study for pepper maturity detection, a detailed performance comparison and analysis were conducted with multiple object detection models, including Faster-RCNN, SSD, YOLOv5, YOLOv7, YOLOv8n, and YOLOv11. The experimental results show that YOLO-ALW has a significantly lower number of parameters (3.166 M) compared to YOLOv5 (7.24 M) and YOLOv7 (37.49 M). It also demonstrates considerable advantages in terms of floating-point operations (8.2 G) and model size (6.28 MB). Specifically, compared to YOLOv5, the computation load is reduced by 8.4G and the storage requirement is decreased by 7.82 MB. In comparison with YOLOv7, the computation load is reduced by 115.3 G and the storage space is reduced by 68.22 MB. Although YOLO-ALW has a slightly higher number of parameters (3.166 M) than YOLOv11 (2.58 M), and a higher floating-point operation count (8.2 G compared to 6.3 G) and model size (6.28 MB compared to 5.22 MB), it remains competitive in terms of core evaluation metrics.

In terms of precision and recall, YOLO-ALW achieved 98.3% and 97.8%, significantly outperforming YOLOv5 (91.3%, 81.7%) and YOLOv7 (94.2%, 91.6%). Specifically, precision increased by 7% and recall by 16.1% compared to YOLOv5, and precision improved by 4.1% and recall by 6.2% compared to YOLOv7. Although YOLO-ALW slightly lags behind YOLOv11 in precision (96.2%), it surpasses YOLOv11 in recall (97.8% vs. 91.7%). Notably, in mAP0.5, YOLO-ALW achieved 99.1%, surpassing all other models, with improvements of 13.6% and 6.8% over YOLOv5 and YOLOv7, respectively, a 3.4% improvement over YOLOv8n (95.7%), and a 1.5% improvement over YOLOv11 (97.6%). In contrast, SSD and Faster-RCNN performed weakly in the pepper maturity detection task, particularly in terms of precision and recall, where they significantly lagged behind YOLO-ALW. In mAP0.5, SSD and Faster-RCNN were lower than YOLO-ALW by 7.9% and 15.5%, respectively. Overall, while YOLO-ALW shows some trade-offs in specific metrics compared to YOLOv11, its exceptional performance across multiple dimensions robustly demonstrates its efficiency, accuracy, and substantial potential for real-world applications in pepper maturity detection. The experimental results are presented in Table 6.

**Figure 9:**
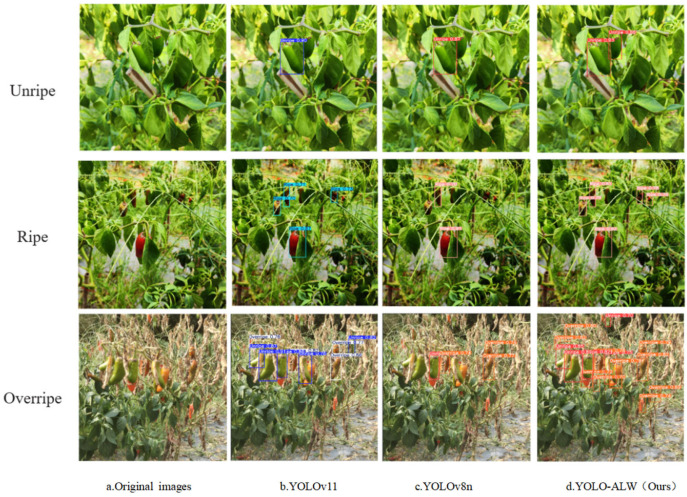
Comparison of model detection results.

We compare the prediction of the three models with the best overall performance in the results of the comparison experiments, and the confidence level is set to 0.7. Figure 9 demonstrates the results of YOLOv11, YOLOv8n, and YOLO-ALW for detecting the ripeness of chili peppers in different scenarios.

In the detection of immature peppers, all three models, YOLOv11, YOLOv8n, and YOLO-ALW, are capable of accurately identifying the location of the peppers, marking them with bounding boxes of different colors and displaying corresponding prediction information. However, a closer inspection reveals that the bounding boxes marked by YOLO-ALW appear to more closely align with the actual contours of the peppers, and the displayed prediction information is both clearer and more accurate. This demonstrates, to some extent, the advantages of YOLO-ALW in detecting immature peppers.

For the detection of ripe peppers, all three models perform relatively well. YOLOv11 is able to quickly locate ripe peppers, but in terms of handling finer details—such as the precision of the bounding boxes and the completeness of the prediction information—it is slightly inferior to YOLO-ALW. YOLOv8n also completes the detection task well, but overall, YOLO-ALW outperforms the other models with its more precise bounding boxes and richer, more accurate prediction information, showcasing higher detection quality and accuracy.

In the detection of overripe peppers, the challenge becomes more significant due to changes in the color and morphology of the peppers. Despite this, YOLO-ALW maintains a high level of detection performance, with the accuracy of its bounding boxes and the reliability of its prediction information standing out among the three models. In contrast, YOLOv11 and YOLOv8n show some slight deviations in bounding box accuracy and prediction information reliability when detecting overripe peppers.

In summary, the detection results shown in Figure 9 highlight that, across different scenarios for detecting pepper maturity, YOLO-ALW demonstrates a clear advantage over YOLOv11 and YOLOv8n in terms of detection accuracy, bounding box precision, and prediction information reliability. This further validates its superior performance and robust capabilities in the field of pepper maturity detection.

Based on the data in Table 7, the processing speeds (images per second, it/s) of YOLO-ALW (the model proposed in this study), YOLOv11, and YOLOv8n are relatively comparable. YOLO-ALW achieves a processing speed of 9.54 it/s, slightly surpassing YOLOv8n at 9.41 it/s, while YOLOv11 has a marginally higher speed of 9.64 it/s. This indicates that YOLO-ALW maintains competitive performance in processing speed, enabling it to handle a substantial amount of image data per unit of time, making it suitable for applications requiring high real-time performance.

Regarding the time consumption in different processing stages, YOLO-ALW and YOLOv11 have the same preprocessing time of 0.3 ms, whereas YOLOv8n’s preprocessing time is longer at 0.6 ms. Although preprocessing time typically accounts for a small proportion of the overall processing pipeline, such minor differences may accumulate and affect efficiency in large-scale data processing. The inference time, which is a key factor affecting response speed, is longest for YOLOv8n (3.9 ms), followed by YOLO-ALW (3.1 ms) and YOLOv11 (2.8 ms). Although YOLO-ALW does not have the shortest inference time, it still maintains a competitive edge in overall processing speed, suggesting that its structural design or resource allocation contributes to improved efficiency. The postprocessing time is 0.8 ms for both YOLO-ALW and YOLOv11, while YOLOv8n is slightly longer at 0.9 ms. Although this difference is small, it should be considered in applications that demand optimal real-time performance.

In conclusion, YOLO-ALW demonstrates notable advantages in real-time performance, maintaining competitive processing speeds and exhibiting a balanced time distribution across different processing stages. However, real-time performance optimization remains an ongoing task, and future research could focus on further refining the model structure and algorithms to enhance its performance in various real-world applications.

The experimental results demonstrate that the YOLO-ALW model exhibits exceptional overall performance across all categories, with a precision of 98.3%, recall of 97.8%, and an impressive mAP_0.5_ of 99.1%. Furthermore, the model effectively balances precision and recall in classifying peppers at different maturity stages, highlighting its significant potential for practical applications. With further optimization of the training and testing data, it is anticipated that the model’s performance will improve in more complex backgrounds and broader application scenarios. The experimental results are presented in Table 8.

#### 3.4.3. Comparison of Model Feature Visualization

**Figure 10:**
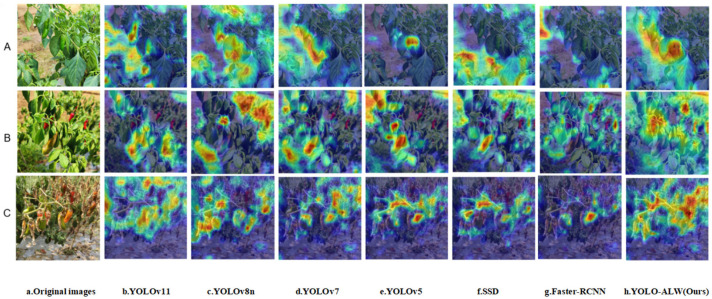
Comparison of model heatmap results.

Grad-CAM (gradient-weighted class activation mapping) [41] is a visualization technique used to identify the regions of an image that a deep learning model attends to during classification tasks. It operates by analyzing the model’s gradients to determine which input features are most influential in predicting a specific class. In this study, a confidence threshold of 0.65 was applied to generate visual heatmaps. In these heatmaps, the brightness of the colors corresponds to the intensity of the model’s response in the respective regions. Brighter regions indicate a higher influence of those areas on the model’s prediction. The resulting heatmaps are shown in Figure 10.

As observed from Figure 10, the enhanced YOLO-ALW model exhibits superior performance in detecting the maturity features of peppers, demonstrating improved adaptability and robustness across the diverse scenarios depicted in subfigures A–C. In subfigure A, which represents scenes with similar backgrounds, the model accurately identifies peppers, effectively mitigating misclassifications caused by background interference. In subfigure B, illustrating complex environments with multiple disturbances, the model maintains precise localization and recognition of the peppers, showcasing its resilience to challenging conditions. In subfigure C, depicting multi-object scenarios, the model efficiently identifies and localizes multiple peppers simultaneously, even amidst significant clutter. These results indicate that the improved YOLO-ALW model generates higher heatmap response values during the pepper maturity detection task, reflecting its heightened sensitivity to subtle variations in pepper maturity features. This capability is essential for accurate maturity classification and offers substantial application potential, particularly in precision agriculture, quality control, and other fields demanding high-accuracy recognition.

## 4. Discussion

The YOLO-ALW model introduced in this study marks a notable progression in the domain of pepper maturity detection, addressing several critical gaps in the existing literature and offering substantial contributions to the field. Below, we discuss how this study advances the state of the art in chili pepper maturity detection and how it addresses the limitations of prior research.

### 4.1. Addressing Labor and Resource Intensity in Traditional Methods

In the context of pepper cultivation, the labor intensity of traditional chili maturity detection is considerable. Manual inspection, the primary method, requires workers to visually assess each pepper individually. In large-scale pepper farms, this process is time-consuming and physically demanding. For instance, in a medium-sized plantation with 50,000 plants, assuming each worker can inspect approximately 500 peppers per hour, a team of 20 workers would require around 50 h to complete the inspection. This not only leads to worker fatigue but also hinders the overall harvesting efficiency. Additionally, as the day progresses, fatigue may result in decreased accuracy in maturity assessment, increasing the likelihood of harvesting underripe or overripe peppers.

The implementation of the YOLO-ALW model has the potential to significantly transform the chili maturity detection process. By integrating the model into automated imaging systems on harvesting machinery or drones, the need for manual inspection can be substantially reduced. In a medium-sized plantation, an automated system utilizing the YOLO-ALW model could scan the entire plantation in just a few hours. For example, a drone equipped with a high-resolution camera and the model could cover a large area quickly, analyzing thousands of peppers per minute. This would enable workers to focus on other critical tasks, such as sorting and packaging, thus optimizing the overall harvesting process. Moreover, the model’s high precision in detection ensures that only peppers at the optimal maturity stage are harvested, enhancing the quality of the produce and increasing the economic returns for farmers.

From the perspective of labor intensity, traditional chili maturity detection primarily relies on manual observation, which demands substantial human labor. In large-scale cultivation settings, workers must inspect each pepper individually to assess its maturity, a process that is not only time-consuming and physically demanding but also prone to errors due to fatigue. The application of the YOLO-ALW model automates the maturity detection process. By integrating the model into smart harvesting equipment or imaging systems, devices can quickly capture and analyze pepper images, significantly reducing the need for manual inspections. For instance, during peak harvesting periods, the time required for manual detection is substantially reduced, allowing workers to focus on other critical aspects of agricultural production.

Regarding resource intensity, the YOLO-ALW model also offers advantages. Compared to more complex deep learning models, YOLO-ALW has fewer parameters (3.166 M) and lower floating-point operations (8.2 G). This results in reduced computational resource requirements, enabling efficient operation on standard hardware, such as low-cost edge computing devices. This not only lowers hardware acquisition costs but also reduces energy consumption. Additionally, the model’s lightweight design minimizes data storage needs, as smaller model files occupy less storage space, further decreasing resource intensity.

### 4.2. Novel Contributions to Model Enhancement

With respect to model enhancement, prior research [1,2,4,5,19,27] has employed various approaches for fruit maturity detection, but often encounters challenges related to the complex morphology and occlusion of peppers in imaging contexts. For instance, Lin et al. [1] developed a model for tobacco leaf maturity, which is not directly translatable to peppers due to the distinct morphological characteristics of the two plants. Similarly, Agung et al. [2] demonstrated high accuracy in detecting chili pepper maturity, but their method may lack robustness in the presence of occlusion. Li et al. [27] proposed the integration of YOLOv4-Tiny with an adaptive spatial feature pyramid and the CBAM attention mechanism to improve detection of occluded and small targets. However, as our study highlights, their approach remains limited when faced with complex background conditions. In contrast, our introduction of the AKConv module offers a novel solution by adaptively adjusting convolutional kernels to better capture pepper features in dense and partially occluded scenarios, representing a significant advancement over traditional methods using fixed convolutional kernels [37]. Future research could build on Zhang et al.’s work on kernel optimization [37] to refine the AKConv module’s parameter learning process, enabling more rapid and precise adaptation to various occlusion levels and pepper shape variations.

In terms of multi-scale feature integration, earlier studies [18,19,21,22] have attempted to enhance feature extraction in fruit images but often struggle with special textures and lighting conditions. For example, Chen et al. [18] improved the YOLOv7 model for oil tea maturity detection using an attention mechanism, which may not be sufficiently robust for handling the complex textures and lighting variations present in pepper images. Our SPPF_LSKA module, by contrast, enhances multi-scale feature integration by decomposing the traditional 2D convolution kernel, reducing computational complexity while improving the model’s capacity to capture long-range dependencies [38]. Despite these improvements, further work could explore advanced texture analysis techniques, such as those used in [32,33,34] for fruit quality assessment, or apply illumination normalization methods to further enhance feature representation and model robustness in varied environments. This could lead to better discrimination across different pepper maturity levels, addressing a notable gap in the current literature.

Regarding loss functions, traditional Intersection over Union (IoU)-based loss functions, prevalent in many pepper detection studies [27], face limitations in scenarios involving occlusion and backgrounds with similar color properties. The Wise-IoU loss function, introduced in our model, incorporates a dynamic focusing mechanism that enhances gradient allocation, thereby improving performance in challenging scenarios. Our experimental results demonstrate that the Wise-IoU function outperforms traditional IoU-based losses, particularly in small target detection and cases involving color similarity or occlusion [39]. However, challenges remain when dealing with rare background interferences or unconventional pepper arrangements. Future work could build on the ideas from [39] and integrate the Wise-IoU loss function with other loss functions, such as the Generalized Focal Loss v2 proposed by Li et al. [36], to further augment the model’s adaptability and generalization in complex real-world environments.

When compared to other models, lightweight models [28] may achieve faster detection speeds in simpler contexts but often at the expense of accuracy. Our YOLO-ALW model strikes a balance between precision and the ability to handle complex scenarios. In contrast to models like Faster-RCNN and SSD, which performed suboptimally in our pepper maturity detection task, YOLO-ALW shows superior results in terms of precision, recall, and mAP_0.5_ [6,27]. Future research could explore strategies such as model fusion or adaptive model selection, inspired by [40], to dynamically adjust the model architecture based on specific application requirements, improving detection efficiency in pepper maturity assessment.

Finally, many existing studies [1,2,3,4,5,6,18,19,20,21,22,23,24,25,26,27] rely on datasets with limited diversity, often constrained by factors like geographic location and pepper variety. While our dataset incorporates some variability, it too is limited by similar factors. Expanding the geographical and varietal scope of the dataset, as suggested in [28,29], would improve the model’s generalization across diverse agricultural contexts. Additionally, the incorporation of dynamic data throughout the pepper growth process, as proposed by [30] for crop monitoring, would enable the model to capture temporal features associated with pepper maturation, thereby enhancing prediction accuracy and reliability. This approach addresses a significant gap in the literature regarding the use of comprehensive and diverse datasets for pepper maturity detection.

## 5. Conclusions

The YOLO-ALW model proposed in this study achieves high-precision detection of pepper maturity. By improving and optimizing the loss function, backbone, and head components, and incorporating the SPPF_LSKA module, adaptive kernel convolution (AKConv), and Wise-IoU loss function, the model effectively addresses several challenges in pepper detection. These challenges include plant growth with dense foliage causing occlusion, the similarity in color between immature peppers and their background, and the small size and mutual occlusion of clustered fruits. These enhancements contribute to improved localization accuracy, feature extraction capability, and the ability to capture critical features of the peppers.

Ablation experiments demonstrate the synergistic effect of the various modules in enhancing the model’s detection performance. When all modules were integrated into the baseline network, the model achieved optimal performance, with a mean average precision (mAP_0.5_) of 99.1%, and precision and recall values of 98.3% and 97.8%, respectively. These improvements represent increases of 3.4%, 5.1%, and 9.0%, compared to the original YOLOv8n model. Comparative experiments with other object detection models highlight YOLO-ALW’s clear advantages in resource utilization efficiency and model lightweighting, while also demonstrating exceptional performance in terms of accuracy, recall, and mAP_0.5_. This performance underscores the model’s robust ability to handle high-precision object detection in complex scenarios and its significant potential for practical applications.

Feature visualization results show that the improved YOLO-ALW model excels in recognizing key maturity features of peppers. It is capable of more precisely capturing the critical characteristics of pepper fruits, with improved localization accuracy and a heightened ability to focus on and identify key features. This enhancement enables the model to perform rapid and accurate pepper maturity detection in complex environments, meeting the requirements of real-world applications.

## Figures and Tables

**Table 1:** Chili pepper collection quantity.

Weather Conditions	Number of Images Collected	Chili Pepper Varieties
Sunny	344	Plate pepper
Cloudy	334	Chili Pepper
Overcast	347	Chili Pepper
Sunny	431	Plate pepper

**Table 2:** Chili data collection details.

Camera Equipment	Shooting Angle	Pixel Size	Shooting Ratio	Overlap Condition	Lighting Condition
Xiaomi 12	Eye level view	3024 × 3024	1:1	25%	Low light
iPhone 14	Top–down view	4032 × 3024	4:3	50%	Strong light
Huawei P40	Upward view	3000 × 3000	1:1	50%	Low light
Xiaomi 13	Downward view	3072 × 3072	1:1	25%	Strong light

**Table 3:** Criteria for categorizing the maturity of chilis.

Evaluation Criteria	Unripe	Ripe	Overripe
Color	Full but light in color	Full and consistent in inherent color	Dull and inconsistent in color
Texture	Soft fruit with a smooth surface	Smooth surface and firm fruit	Surface with depressions and other damages
Shape	Fruit without defects, upright shape	Straight or curved fruit	Various defect states, and the fruit is prone to bending and depression
Time	0–25 days after pollination	30–50 days after pollination	Over 50 days after pollination
Color Ratio	Green accounts for 90%	Fruit shows red or dark green	Mixed colors appear, and the proportion of mixed colors exceeds 20%
Fruit Size	Less than 30 cm	Greater than 30 cm	Greater than 30 cm

**Table 4:** Model training parameters.

Parameter	Set Value
Model Training Epochs	100
Batch Size	16
Initial Learning Rate	0.01
Weight Decay Coefficient	0.0005
Input Image Resolution	640 × 640
Optimizer	SGD

**Table 5:** Ablation experiment performance comparison.

YOLOv8n	AKConv	SPPF_LSKA	Wise-IoU	mAP_0.5_ (%)	Precision (%)	Recall (%)
√				95.7	93.2	88.8
√	√			97.7	96.9	94.3
√		√		97.3	96.4	94.7
√			√	97.4	96.5	96.9
√	√	√		97.8	96.1	94.9
√	√		√	97.8	96.8	95.3
√		√	√	98.1	97.2	96.3
**√**	**√**	**√**	**√**	**99.1**	**98.3**	**97.8**

**Table 6:** Comparison of different models’ experimental results.

Models	Parameters/M	FLOPs/G	Model Size/MB	mAP_0.5_ (%)	Precision (%)	Recall (%)
YOLOv5	7.24	16.6	14.1	85.5	91.3	81.7
YOLOv7	37.49	123.5	74.5	92.3	94.2	91.6
YOLOv8n	3.006	8.1	5.9	95.7	93.2	88.8
YOLOv11	2.58	6.3	5.22	97.6	96.2	91.7
SSD	26.29	62.8	93.3	91.2	93.4	73.1
Faster-RCNN	137.10	370.2	111.5	83.6	67.8	81.6
**YOLO-ALW (Ours** **)**	**3.166**	**8.2**	**6.28**	**99.1**	**98.3**	**97.8**

**Table 7:** Real-time performance comparison of models.

Model	Processing Speed (it/s)	Preprocessing Time (Per Image)	Inference Time (Per Image)	Postprocessing Time (Per Image)
YOLOv11	9.64	0.3 ms	2.8 ms	0.8 ms
YOLOv8n	9.41	0.6 ms	3.9 ms	0.9 ms
**YOLO-ALW (Ours** **)**	**9.54**	**0.3 ms**	**3.1 ms**	**0.8 ms**

**Table 8:** YOLO-ALW modeling results on different pepper maturity levels.

Class	Precision (%)	Recall (%)	mAP_0.5_ (%)
All	0.983	0.978	0.991
Unripe	0.988	0.992	0.995
Ripe	0.984	0.962	0.984
Overripe	0.979	0.981	0.994

## Data Availability

Data are available on request due to privacy.
